# QTLs of factors of the metabolic syndrome and echocardiographic phenotypes: the hypertension genetic epidemiology network study

**DOI:** 10.1186/1471-2350-9-103

**Published:** 2008-11-27

**Authors:** Aldi T Kraja, Pinchia Huang, Weihong Tang, Steven C Hunt, Kari E North, Cora E Lewis, Richard B Devereux, Giovanni de Simone, Donna K Arnett, Treva Rice, DC Rao

**Affiliations:** 1 Division of Statistical Genomics, Washington University School of Medicine, Saint Louis, MO, USA; 2 Division of Biostatistics, Washington University School of Medicine, Saint Louis, MO, USA; 3 Division of Epidemiology and Community Health, University of Minnesota, Minneapolis, MN, USA; 4 Divison of Cardiovascular Genetics, University of Utah School of Medicine, Salt Lake City, Utah, USA; 5 Department of Epidemiology, University of North Carolina, Chapel Hill, North Carolina, USA; 6 Department of Medicine, University of Alabama at Birmingham, Birmingham, AL, USA; 7 Department of Medicine, Weill Cornell Medical College, New York, NY, USA; 8 Department of Clinical and Experimental Medicine, Federico II University, Naples, Italy; 9 Department of Epidemiology, University of Alabama at Birmingham, Birmingham, USA

## Abstract

**Background:**

In a previous study of the Hypertension Genetic Epidemiology Network (HyperGEN) we have shown that metabolic syndrome (MetS) risk factors were moderately and significantly associated with echocardiographic (ECHO) left ventricular (LV) phenotypes.

**Methods:**

The study included 1,393 African Americans and 1,133 whites, stratified by type 2 diabetes mellitus (DM) status. Heritabilities of seven factor scores based on the analysis of 15 traits were sufficiently high to pursue QTL discovery in this follow-up study.

**Results:**

Three of the QTLs discovered relate to combined MetS-ECHO factors of "blood pressure (BP)-LV wall thickness" on chromosome 3 at 225 cM with a 2.8 LOD score, on chromosome 20 at 2.1 cM with a 2.6 LOD score; and for "LV wall thickness" factor on chromosome 16 at 113.5 with a 2.6 LOD score in whites. The remaining QTLs include one for a "body mass index-insulin (BMI-INS)" factor with a LOD score of 3.9 on chromosome 2 located at 64.8 cM; one for the same factor on chromosome 12 at 91.4 cM with a 3.3 LOD score; one for a "BP" factor on chromosome 19 located at 67.8 cM with a 3.0 LOD score. A suggestive linkage was also found for "Lipids-INS" with a 2.7 LOD score located on chromosome 11 at 113.1 cM in African Americans. Of the above QTLs, the one on chromosome 12 for "BMI-INS" is replicated in both ethnicities, (with highest LOD scores in African Americans). In addition, the QTL for "LV wall thickness" on chromosome 16q24.2-q24.3 reached its local maximum LOD score at marker D16S402, which is positioned within the 5th intron of the *cadherin 13 *gene, implicated in heart and vascular remodeling.

**Conclusion:**

Our previous study and this follow-up suggest gene loci for some crucial MetS and cardiac geometry risk factors that contribute to the risk of developing heart disease.

## Background

Metabolic Syndrome (MetS), a cluster of obesity, insulin resistance and glucose intolerance, dyslipidemia, and high blood pressure, is related to echocardiographic (ECHO) measurements of the heart. For example, left ventricular hypertrophy (LVH) is a complex trait that is a major manifestation of target organ damage in hypertension [1]. MetS and LVH are reported to increase the risk of cardiovascular (CV) disease [2-6]. In a recent study we further explored the relationships among these traits by utilizing multivariate factor analysis (FA). Correlations among 15 metabolic and echocardiographic traits analyzed showed significant relationships among MetS risk factors (especially systolic and diastolic blood pressure (BP) and body mass index (BMI)) and cardiac phenotypes. Factors identified represented new combined MetS-ECHO domains as for example "BP-LV geometry," and "BP-LV wall thickness," and also represented known domains in the MetS such as "BMI-INS," "Lipids-INS," "BP," and ECHO domains "LV wall thickness," and "LV geometry." Quantitative trait loci (QTLs) discovery was warranted based on the heritability estimates reported [7].

Until recently, different studies have reported QTLs for MetS or ECHO. Teran-Garcia and Bouchard [8] provide a comprehensive review of QTLs associated with MetS. In one of their cited studies, Kraja et al [9] studied QTLs for MetS factors in the HyperGEN study for two ethnicities. A QTL with logarithm of odds (LOD) score of 2.8 on chromosome 13p12 for the obesity-INS factor and one with a LOD of 2.6 on chromosome 11q24 for the lipids-INS factor were described for African Americans. Also, QTLs for the BP factor (LOD of 3.2 on chromosome 15q15), for the lipids-INS factor (a LOD of 3.08 on chromosome 8p23), and for the obesity-INS factor (LOD of 3.1 on chromosome 3p26) were reported in whites. More recently both linkage and association analysis of ECHO traits have been reported in the HyperGEN study. Arnett et al [10] studying the LV contractility, reported a LOD of 3.9 at 54 cM on chromosome 11 in African Americans and a 2.8 LOD score at 17.9 cM on chromosome 22. Tang et al [11] reported QTLs for LV early diastolic peak E velocity on chromosome 5 at 133.6 cM with a LOD of 3.6 in African Americans, and a LOD score of 2 on chromosome 12 at 105–109 cM for peak A velocity in whites. In the third paper, Bella et al [12] studied linkage for valve calcification finding LOD scores of 3.2 and 2.6 respectively at 105.6 and 130.4 cM on chromosome 16, and a LOD of 2.9 at 48 cM of chromosome 19.

Another recent publication of Mayosi et al [13] performed genome wide linkage analysis on LV mass of 826 subjects of British families ascertained for high BP, by selecting the proband from the top 5% of the population distribution. They obtained moderate LOD scores (1.5–2.67) for electrocardiographic and echocardiographic LV mass.

The present study extends previous investigations by focusing concurrently on both MetS risk components and ECHO phenotypes. In addition, we expect an increase in power for QTL discovery by utilizing FA factors, because they capture inter-variable correlations. In this study we aimed to identify QTLs that control genetic variability of combined MetS-ECHO factors, as well as individual MetS and ECHO factor domains in the Hypertension Genetic Epidemiology Network Study (HyperGEN).

## Methods

### Subjects

HyperGEN is part of the NHLBI Family Blood Pressure Program (FBPP), which studies the genetic aspects of high blood pressure and related conditions [14]. Further details for the HyperGEN study are provided elsewhere [7,9-12]. In the current study the focus was on the siblings and the offspring of the sampled populations. Subjects with less than 8 hours of fasting time, or missing values for any of the 15 traits studied were excluded from the analysis. Consequently, in our study the phenotypes of 1,393 African Americans and of 1,133 Caucasians were analyzed. The samples were organized also by including/excluding diabetes mellitus (DM) subjects (for more details see Huang et al [7]). Clinical data, echocardiographic measurements, questionnaires, and blood samples were collected with informed consent from the participants.

### Echocardiography

Echocardiograms followed standard protocols as described by Devereux and Roman [15] to obtain two-dimensional (2D) guided M-mode, 2D and Doppler measurements. The measurements were first made by sonographers or physicians centrally trained at the Reading Center in New York and subsequently verified or changed by highly experienced cardiologists in the Reading Center, who were blinded to clinical data.

### Phenotypic Data

The 15 traits that follow constitute the original traits which through multivariate factor analyses were transformed into factor scores. The factor scores served as phenotypes in our linkage analyses. Fasting glucose (GLUC) and insulin (INS); triglycerides (TG) and high-density lipoprotein cholesterol (HDLC); body mass index (BMI); medication-adjusted systolic and diastolic blood pressure (SBP and DBP); heart rate (HR), diastolic LV internal dimension (LVID), posterior wall thickness (PWT) and relative wall thickness (RWT); LV mass indexed to height^2.7 ^(LVMI), aortic root diameter (ARD), arterial stiffness defined by pulse pressure/stroke volume (PP/SV), and LV midwall shortening (MWS) underwent appropriate distribution transformations and removal of only a few outliers to achieve normality. Log transformation was applied to INS, HDLC, TG, BMI, RWT, LVMI, PWT, and PP/SV; the reciprocal of squared GLUC (1/GLUC^2^) and cubic power transformation for MWS (MWS^3^) were applied in both African Americans and whites (see Huang et al. [7] for details). The SBP and DBP of subjects receiving anti-hypertensive medication were adjusted for the anti-hypertensive medication(s) following the method described by Wu et al. [16] and applied in the HyperGEN study to estimate untreated BP levels for various classes of medications. Stepwise regression using the *REG *procedure of SAS was used for the covariate adjustments for each of 15 variables, within race and gender by regressing on age, age^2^, age^3 ^and field centers, and retaining only the significant terms. Age and field center effects were removed from the means and the variances, and standardized residuals were derived. A few outliers beyond ± 4 standard deviations and greater than 1 standard deviation away from the next internal data point were eliminated. Final standardized residuals were utilized in the FA. FA is a multivariate statistical technique which explains variability of primary variables in terms of fewer unobserved (latent) variables named factors. The latent factors help to identify inter-correlations among primary variables. Two categories of factor analysis, with and without Varimax rotation, were performed, as previously described [7]. FA without rotation achieves the simplest factor structure, where the loading of a variable is maximum with one factor, associated with little contribution of that variable to other factors. In contrast, Varimax rotation identifies factors representing distinct clusters of interrelated variables. At least two risk variables in a latent factor were required with loadings of about 0.4 or greater. The factor loadings were essentially the correlation coefficients between each original variable and the latent factor. Factor loadings represent the contribution of each original variable to the latent factor. The original variables contribution into factors helped to label each latent factor. The squared factor loading represented the percentage of variance in the original variable associated with the latent factor. The sum of squared loadings per latent factor had to approach or exceed one and the maximum likelihood estimate model was required to be significant (p-value < 0.05). Factor analysis applied on the 15 normally distributed traits, produced factor scores which finally were employed in linkage analyses.

### Linkage analysis

The HyperGEN study genotyped 391 microsatellite markers at the National Heart, Lung, and Blood Institute Mammalian Genotyping Service (Marshfield, WI), of which 370 autosomal markers were used for this genome-wide linkage analysis. Software used to insure the marker' quality control included ASPEX, GRR, GENEHUNTER and PEDCHECK [17-19]. The average inter-marker distance was about 10 centiMorgans (cM). The gender-averaged genetic distances were retrieved from the Marshfield human genetic linkage map. Ethnic-specific allele frequencies were calculated based on the random sample included in the HyperGEN study. Pedigrees were split in nuclear families for analyses. Multipoint identity by descent coefficients were estimated with MAPMAKER/SIBS software, at 1 cM contiguous distances [18]. Finally, a non-parametric variance component linkage analysis was performed via SEGPATH software [20]. The non-parametric (or model free) linkage method applied does not depend on the assumptions about the penetrance of the phenotype. On the other hand the marker locus model is applied by hypothesizing an additive effect of a QTL (g), a residual polygenic component (G_R_), and a residual non-familial variance component (r). The null hypothesis of no linkage was tested by restricting the QTL heritability (h^2^_g_) at the putative locus at h^2^_g _= 0. The linkage test becomes a likelihood ratio of the alternative hypothesis against the null hypothesis, in every location throughout the genome where h^2^_g _is estimated. This test turns out to be a χ^2 ^with 1 degree of freedom [20].

## Results

Table 1 for the African American's sample is a summary of LOD scores greater than 1.7 [see also the Additional files 1, 2, 3, 4]. Significant LOD scores greater than or close to 3 were found for "BP" domain in the African American sample in all data for FA with Varimax rotation (LOD score of ~3.0, with a peak at 67.8 cM distance on chromosome 19). QTLs for "BMI-INS" factor domain were identified when DM subjects were excluded (LOD of 3.9, at 64.8 cM on chromosome 2, and LOD of 3.3 at 91.4 cM on chromosome 12). For the same ethnicity, when FA with NO rotation was applied in all data, the above findings of chromosomes 2 and 19 replicated with LOD scores respectively of 2.8 and 2.7. For FA with NO rotation, when excluding DM participants two peaks for "BMI-INS" domain replicated above significant LOD scores (on chromosome 2, at 65.8 cM, LOD of 3.6, and on chromosome 12, at 91.4 cM, LOD of 3.2).

**Table 1:** Major QTL findings per chromosome, LOD > 1.7 in African Americans

No	Rotation	Excluding DM	Chromosome	Sex-averaged distance in cM, peak and (surrounding markers)	Marker name	Dname	LOD* Score	Factor Domain
1	NO	NO	2	68.76 (64.29–73.61)	ATA4F03-ATA27D04	D2S1356-D2S1352	**2.84**	BMI-INS
2			12	100.42 (95.03–104.12)	GATA63D12-GATA85A04	D12S1064-D12S1300	1.98	BMI-INS
3			19	68.84 (68.08)	MDF139	D19S178	2.67	BP
4			20	4.13 (2.13)	AFM077XD3	D20S103	1.78	BMI-INS
			
5	NO	YES	2	49.76 (47.97–55.51)	GATA8F07-GATA86E02	D2S405-D2S1788	2.41	BMI-INS
6			2	65.76 (64.29–73.61)	ATA4F03-ATA27D04	D2S1356-D2S1352	**3.62**	BMI-INS
7			5	114 (114.75)	ATA4D10	D5S1453	2.36	BP
8			11	91.11 (85.48–100.82)	GATA30G01-GATA28D01	D11S2002-D11S2000	1.80	BMI-INS
9			12	91.42 (83.19–95.03)	GATA26D02-GATA63D12	D12S1052-D12S1064	**3.22**	BMI-INS
10			18	54.84 (54.40)	GATA64H04	D18S877	2.37	BMI-INS
11			19	70.84 (68.08–78.08)	MFD139-MFD232	D19S178-D19S246	2.18	BP
			
12	VAR†	NO	5	36.00 (36.25)	GATA134B03	D5S2845	2.42	LIPIDS-INS
13			10	153.32 (148.17–165.27)	GGAA5D10-GGAA23C05	D10S1213-D10S1248	1.87	LIPIDS-INS
14			11	113.11 (113.13)	GATA23606	D11S1998	**2.66**	LIPIDS-INS
15			19	67.84 (68.08)	MFD139	D19S178	**2.97**	BP
			
16	VAR	YES	2	64.76 (64.29)	ATA4F03	D2S1356	**3.90**	BMI-INS
17			5	115.00 (115.75)	ATA4D10	D5S1453	2.02	BP
18			12	91.42 (83.19–95.03)	GATA26D02-GATA63D12	D12S1052-D12S1064	**3.34**	BMI-INS
19			18	54.84 (54.40)	GATA64H04	D18S877	2.02	BMI-INS
20			19	68.84 (68.08)	MFD139	D19S178	1.98	BP

* Logarithm of odds; †Varimax rotation

Table 2 is a summary of QTL findings for the white sample [see also the Additional files 5, 6, 7, 8]. The "BP-LV wall thickness" factor, a combination of MetS-ECHO domains, result of FA with NO rotation when excluding DM subjects in whites, showed suggestive linkage with a maximum peak of 2.8 LOD score on chromosome 3 at 224.9 cM, and a 2.6 LOD score on chromosome 20 at 2.1 cM. In addition, in the same ethnicity, for the "LV wall thickness" factor domain, two suggestive peaks were found; one on chromosome 3 at 114.3 cM, with LOD score of 2.7 for all data and NO rotation, and one on chromosome 16 at 113.5 cM, with LOD score of 2.8 when performing Varimax rotation and excluding DM subjects.

**Table 2:** Major QTL findings per chromosome, LOD > 1.7 in whites

No	Rotation	Excluding DM	Chromosome	Sex-averaged distance in cM, peak and (surrounding markers)	Marker name	Dname	LOD* Score	Factor Domain
1	NO	NO	3	114.32 (112.42)	GATA128C02	D3S4529	**2.71**	LV WALL THICKNESS
2			16	113.46 (113.52)	AFM031XA5	D16S402	1.79	LV WALL THICKNESS
3			20	4.13 (2.13)	AFM077XD3	D20S103	1.82	BP-LV WALL THICKNESS
			
4	NO	YES	3	201.32 (201.14)	AFM059XA9	D3S1262	1.98	BP-LV GEOMETRY
5			3	224.88	AFM254VE1	D3S1311	**2.79**	BP-LV WALL THICKNESS
6			12	98.42 (95.03)	GATA63D12	D12S1064	2.32	BMI-INS
7			15	78.30 (82.84)	ATA28G05	D15S655	1.90	LV WALL THICKNESS
8			16	113.46 (113.52)	AFM031XA5	D16S402	**2.61**	LV WALL THICKNESS
9			20	2.13	AFM077XD3	D20S103	**2.61**	BP-LV WALL THICKNESS
			
10	VAR†	NO	3	5.32 (5.54)	GATA22G12	D3S2387	2.01	BMI-INS
11			3	114.32 (112.42)	GATA128C02	D3S4529	2.18	LV WALL THICKNESS
12			11	17.11 (8.9)	ATA33B03	D11S2362	1.81	BMI-INS
13			12	47.42 (48.7)	ATA27A06	D12S1042	1.99	LV GEOMETRY
14			16	113.46 (113.52)	AFM031XA5	D16S402	1.72	LV WALL THICKNESS
15			21	19.99 (24.73)	GATA129D11	D21S2052	1.81	LV WALL THICKNESS
			
16	VAR	YES	3	218.32 (215.84)	ATA22E01	D3S2418	2.26	LV GEOMETRY
17			11	28.11 (33.02)	ATA34E08	UNKNOWN	1.72	BMI-INS
18			12	87.42 (95.03)	GATA63D12	D12S1064	2.00	BMI-INS
19			14	107.46 (105.53)	GGAA21G11	D14S617	1.87	BP
20			16	113.46 (113.52)	AFM031XA5	D16S402	**2.81**	LV WALL THICKNESS

* Logarithm of odds; †Varimax rotation

## Discussion

In the present study we examined a group of latent factors identified by performing factor analyses on 15 MetS or ECHO traits. The seven identified latent factors reduced the complexity of this large number of phenotypes. "Lipids-INS" factor was mainly contributed to by HLDC, TG and INS. "BMI-INS" was another factor identified from MetS domains. From the ECHO variables two latent factors were identified, "LV wall thickness" with main contributions from LVMI, PWT, RWT and MWS, and "LV geometry" with main contributors LVMI, LVID and RWT. "BP" factor, primarily a combination of SBP and DBP, was strongly evidenced in African Americans. However, when factor analysis with no rotation was applied in the whites, BP combined with "LV wall thickness" and "LV geometry" to form two new latent variables "BP-LV geometry" a combination of SBP, DBP, LVMI, PWT and LVID and "BP-LV dimension wall thickness" a combination of SBP, DBP, LVID, RWT and PP/SV. We look at these factors as MetS, ECHO, and a combination of MetS-ECHO latent factors. The presence of BP as a connector between MetS and ECHO is consistent with the increased risk of CV disease associated with hypertension. Chinali et al [21] found that abnormal LV geometry and function are related to the MetS, with increased BP being the MetS component most strongly associated with cardiac abnormalities [22]. Therefore, combined MetS-ECHO domains with BP contributions in the HyperGEN whites extend previous findings.

Two QTLs, one from MetS domains, and one from ECHO were prominent. The chromosome 12 QTL for the "BMI-INS" factor was replicated across ethnic groups, although the LOD scores were larger in African Americans. Its location coincides with linkage reports at 12q24.2 for non-insulin dependent DM, rheumatoid arthritis, and multiple sclerosis [23,24]. In this area the *ACACB *gene located on 12q24.1 may be a candidate. This gene reported in murine studies, has been implicated in controlling mitochondrial fat oxidation and is considered a regulator of energy expenditure [25]. Although Wilson et al [26] reported a finding for fat mass in 12q24, their peak location is about 30–35 cM apart from our "BMI-INS" peak finding.

Of particular note are the results in the region around the marker AFM031XA5 (D16S402) at 16q24.2-q24.3 for the "LV wall thickness" in whites. This location marked by D16S402, has been found to be linked with cadherin 13 (*CDH13*) gene. *CDH13*, which is also called heart *cadherin*, is believed to be a calcium dependent mediator of cell-cell interaction in the heart and acts as negative regulator of neural cell growth. Joshi et al [27] claimed *cadherin *may have multiple signaling functions in vascular remodeling and may protect endothelial cells from oxidative stress-induced apoptosis. The *CDH13 *gene is about 1.2 M bp long, has 14 exons and encodes for a protein with 713 amino acids. This gene is most highly expressed in the heart. The AFM031XA5 marker represents a sequence between 81,851,181–81,851,356 (bp), precisely in the non-coding 5^th ^intronic region. We hypothesize that the *CDH13 *polymorphism, involved in calcium mediated cell-cell adhesion, can influence calcium deposition in the heart. Such a hypothesis is supported also by the Bella et al [12] study of valve calcification in the HyperGEN where a significant QTL (3.2 LOD score) was located on chromosome 16 relatively close to the above marker. In contrast, Mayosi et al [13] findings on chromosome 12 and 16 on LV mass do not overlap with our findings. We have a putative QTL located at 47 cM (1.99 LOD score) on chromosome 12 for LV Geometry factor (with contributions of LVID (+), LVMI (+) and RWT (-)) in whites. Theirs, (2.19 LOD score) is located at 75 cM based on the Marshfield genetic map. Our finding on chromosome 16 for LV wall thickness in whites (with contributions of LVID (+), RWT (+), and LVMI (+)) reaches a local maximum of 2.81 LOD score at 113 cM. Theirs is located at the start of chromosome 16 (18.07 cM based on the Marshfield map), and reached a local maximum of 1.85 LOD score. Such differences may arise for several reasons; our study is ascertained for hypertension [14], larger in sample, our factor scores are composite traits compared to theirs for LV mass, to mention a few.

Other genetic linkage findings of interest included the MetS and ECHO factors, for "BP-LV wall thickness" and "BP-LV geometry" in whites (Table 2). For the "BP-LV geometry" factor a QTL on chromosome 3 at marker D3S1262 (201.3 cM) showed a LOD of 2.0 in the same location where an abdominal obesity MetS QTL was reported. [28] A QTL for "BP-LV wall thickness" factor with peak LOD score of 2.8 located on marker D3S1311 (225 cM), in proximity to *DLG1 *gene which is reported to have a role in the epithelial differentiation and regulation of smooth muscle [29,30]. A LOD score of 2.3 was found for "LV geometry" after Varimax rotation, in a region that includes the *FGF12 *gene (fibroblast growth factor 12) which binds to the C terminus of the cardiac voltage-gated sodium channel Na(v)1.5 and modulates the properties of the channel [31].

MetS related factors showed potentially important QTLs for African Americans. For example, "BMI-INS" factor showed highly significant results on 2p22-2p21 and on 12q24.2, as did the "BP" factor on 19q13.1 (Table 1). Chen et al [32] have previously reported strong linkage evidence in West Africans for percent body fat on chromosome 2 at 72.6 cM, relatively close to our 2p22-2p21 QTL finding. A possible candidate gene located at 2p21, close to the position of our linkage markers, is the LSL gene, which controls leptin serum levels. Candidate regions linking to our chromosome 19 BP QTL region have also been reported as for example by Bielinski et al. [33] for pulse pressure. Also Cooper et al [34] described an SBP QTL for Nigerians marked with D19S246 at 78.1 cM (about 7 cM distant from our finding) on the Marshfield map. Finally, in our study LOD scores increased when excluding DM subjects, which can be a reflection of the effect of genetic heterogeneity caused by diabetes.

Our new findings differ in several regards with the previous analysis of MetS in the HyperGEN study [9]. First, the majority of the selected risk factors differ. The previous study had 11 risk variables to characterize only MetS; while the current one includes MetS variables and variables that additionally embrace cardiac geometry and function. Second, the sample sizes are not the same, and vary because of elimination due to missing values per variable and inability to include subjects from the 5^th ^HyperGEN center that did not participate in the ECHO study. Third, only 50–57% of the total original risk factors' variance was explained by the latent factors accepted in the model. Finally, the identified factors differ across studies in terms of the contribution of specific risk factors. Nevertheless when it comes to the "Lipids-INS" factor, (where a key contribution in African Americans was from HDLC, TG, and INS), the suggestive QTL found in the previous study on chromosome 11q24, is replicated in this study with a similar LOD score, but with a shift of the peak location (from 131.3 cM to 113.1 cM). Combined traits analysis can discover QTL locations that affect more than one trait, i.e. with pleiotropic effects. The 11q23-24 human genome location is well known for a cluster of genes (*APOA1, APOC3, APOA4*, and *APOA5*), which effect the lipid levels as well as is associated with DM and heart disease [35]. In contrast, the use of two methodologies with and without Varimax rotation it might artificially increase the chances to obtain significant linkage results. Rao and Gu [36] showed that for a set of 400 genome wide markers, if the LOD score threshold is relaxed to ~1.75, one expects a tolerance of 1 false-positive per genome. It is expected that this threshold, used by us in reporting results, may achieve a better "balance" of the types of the statistical errors. However, in this study we emphasized LOD score results at the level of above 2.5 and 3. Also the arrangements of risk factors into MetS (and ECHO) factors lessen multiple comparison issues [37]. Another problem is the fact that we did not have a full evidence of replication about the QTLs in African-Americans and whites. Hirschhorn et al [38] have shown through simulations that a QTL explaining 20% of variance can produce strong signal in one linkage analysis, but it can be undetectable in another one, simply for the reason of sampling variation. An additional possibility is that a common causal genetic mutation in one population might be rare in another population.

We expected not only that factor scores to provide an opportunity to detect pleiotropic gene effects. In addition, when traits closely related such as SBP and DBP form a separate factor they provide an increased power to detect putative QTLs compared with single trait analysis. For example, analyzing separately SBP and DBP produced weak LOD scores in the peak found on chromosome 19. When analyzed with the factor scores in African Americans we found a LOD score of 2.67, 2.18, 2.97, and 1.98 depending on the rotation method used and including or excluding T2D subjects. Nevertheless we do not know if the BP factor findings are true or type I error results. Consequently, further elucidation of the precise location of the causative polymorphisms of the identified linkage peaks is being processed with an abundant number of SNPs.

## Conclusion

This study is the first genome-wide linkage study that utilizes factors scores derived simultaneously both from domains of MetS and of cardiac geometric and functionality phenotypes. They provide some insight into the genetic relationship underlying MetS and cardiovascular traits. Refined SNPs genotyping with a million SNPs platform will soon enhance the ability to discover causative genes of the cardiac- and MetS related QTLs on chromosome 16q24.2-q24.3 that coincided with linkage within *cadherin 13 *gene and the one on 12q24 which replicated in both ethnicities.

## Abbreviations

ARD: aortic root diameter; BP: blood pressure; BMI: body mass index; cM: centiMorgan; CV: cardiovascular disease; DBP: medication adjusted blood pressure; DM: diabetes mellitus; ECHO: echocardiography; FA: factor analysis; GLUC: fasting glucose; HDLC: high-density lipoprotein cholesterol; HR: heart rate; HyperGEN: Hypertension Genetic Epidemiology Network Study; INS: fasting insulin; LOD: lod score; LV: left ventricular; LVH: left ventricular hypertrophy; LVID: diastolic LV internal dimension; LVMI: LV mass indexed to height^2.7^; MetS: metabolic syndrome; MWS: LV midwall shortening; PP/SV: arterial stiffness defined by pulse pressure/stroke volume; PWT: posterior wall thickness; QTL: quantitative trait loci; RWT: relative wall thickness; SBP: medication-adjusted systolic blood pressure; SNPs: single nucleotide polymorphisms; TG: fasting triglycerides.

## Competing interests

The authors declare that they have no competing interests.

## Authors' contributions

All authors contributed equally.

## Pre-publication history

The pre-publication history for this paper can be accessed here:



## Supplementary Material

Additional file 1 **Figure 1**. Linkage analysis of factors in African Americans, all data, no rotation. The graph represents the linkage analysis results. Click here for file

Additional file 2 **Figure 2**. Linkage analysis of factors in African Americans, excluding DM, no rotation. The graph represents the linkage analysis results. Click here for file

Additional file 3 **Figure 3**. Linkage analysis of factors in African Americans, all data, Varimax rotation. The graph represents the linkage analysis results. Click here for file

Additional file 4 **Figure 4**. Linkage analysis of factors in African Americans, excluding DM, Varimax rotation. The graph represents the linkage analysis results. Click here for file

Additional file 5 **Figure 5**. Linkage analysis of factors, whites all data, no rotation. The graph represents the linkage analysis results. Click here for file

Additional file 6 **Figure 6**. Linkage analysis of factors, whites excluding DM, no rotation. The graph represents the linkage analysis results. Click here for file

Additional file 7 **Figure 7**. Linkage analysis of factors, whites all data, Varimax rotation. The graph represents the linkage analysis results. Click here for file

Additional file 8 **Figure 8**. Linkage analysis of factors, whites excluding DM, Varimax rotation. The graph represents the linkage analysis results. Click here for file
