# Supplementary figures and images for: QTLs of factors of the metabolic syndrome and echocardiographic phenotypes: the hypertension genetic epidemiology network study
Source: BMC Med Genet. 2008 Nov 27;9:103. doi: 10.1186/1471-2350-9-103 (PMC2626585; doi:10.1186/1471-2350-9-103)

# Figure 1. Linkage Analysis of Factors in African Americans, All Data, NO Rotation

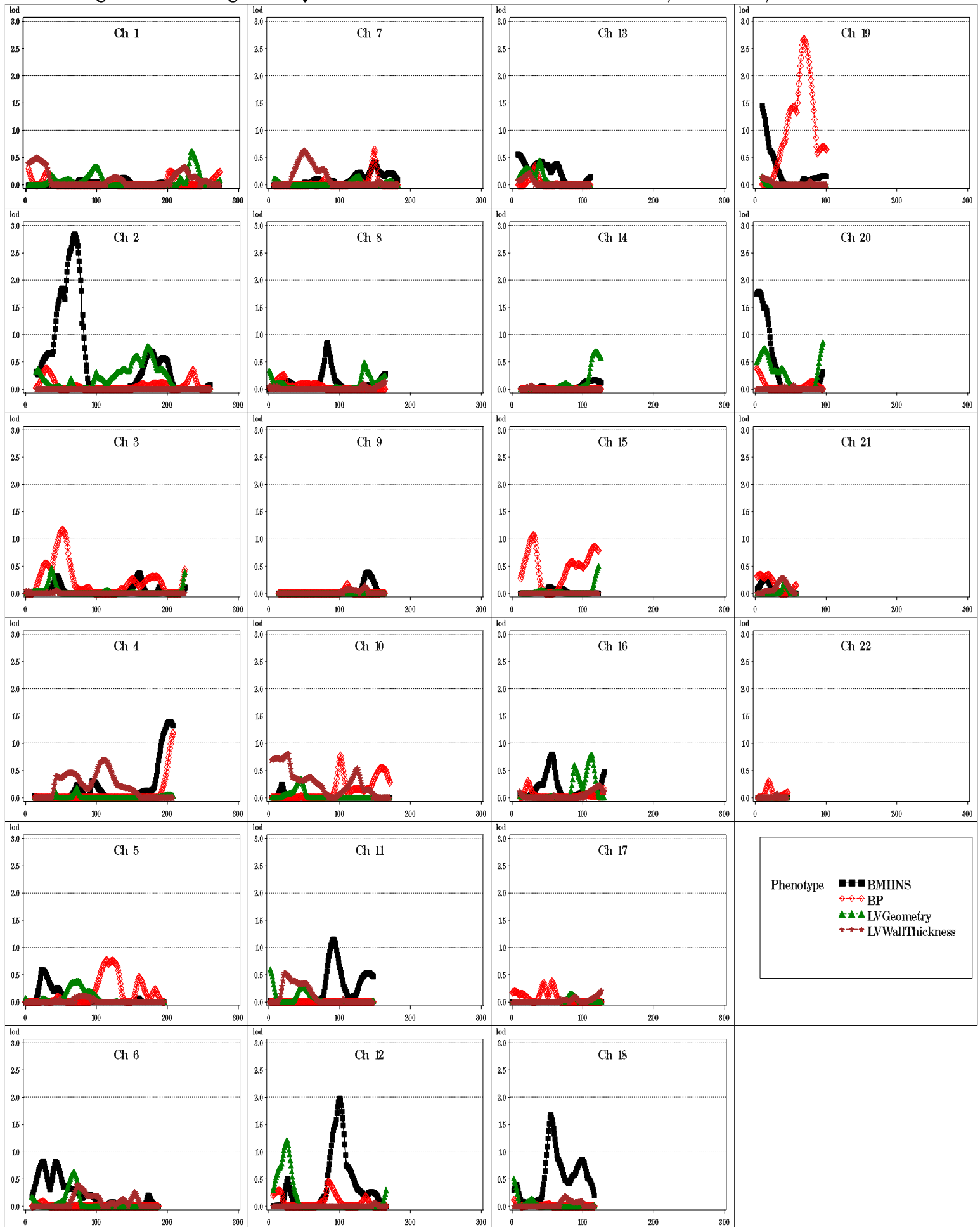

Supplement: Additional file 1 — Figure 1. Linkage analysis of factors in African Americans, all data, no rotation. The graph represents the linkage analysis results. [file 1471-2350-9-103-S1.pdf]

# Figure 2. Linkage Analysis of Factors in African Americans Excluding DM, NO Rotation

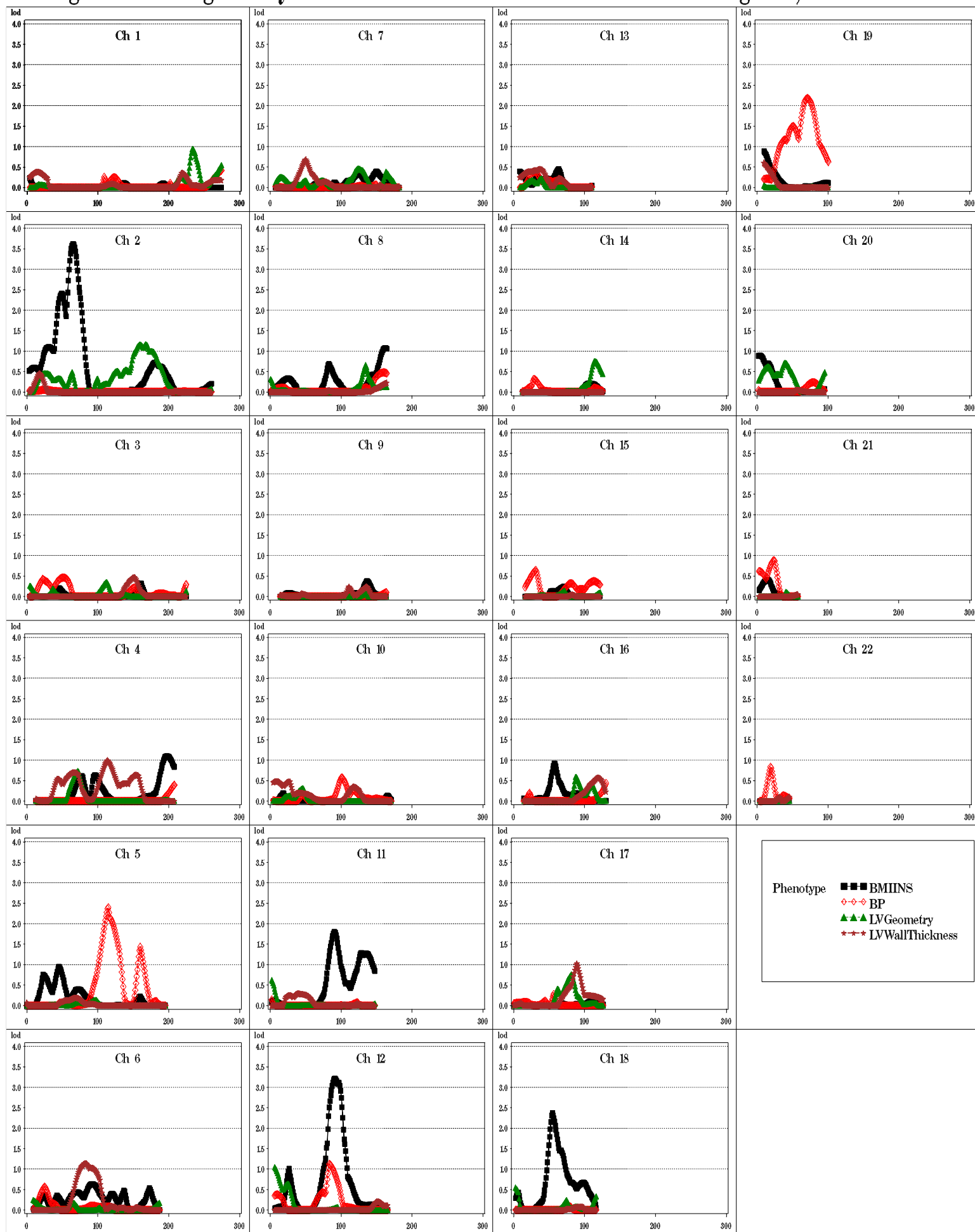

Supplement: Additional file 2 — Figure 2. Linkage analysis of factors in African Americans, excluding DM, no rotation. The graph represents the linkage analysis results. [file 1471-2350-9-103-S2.pdf]

Figure 3. Linkage Analysis of Factors in African Americans, All Data, Varimax Rotation

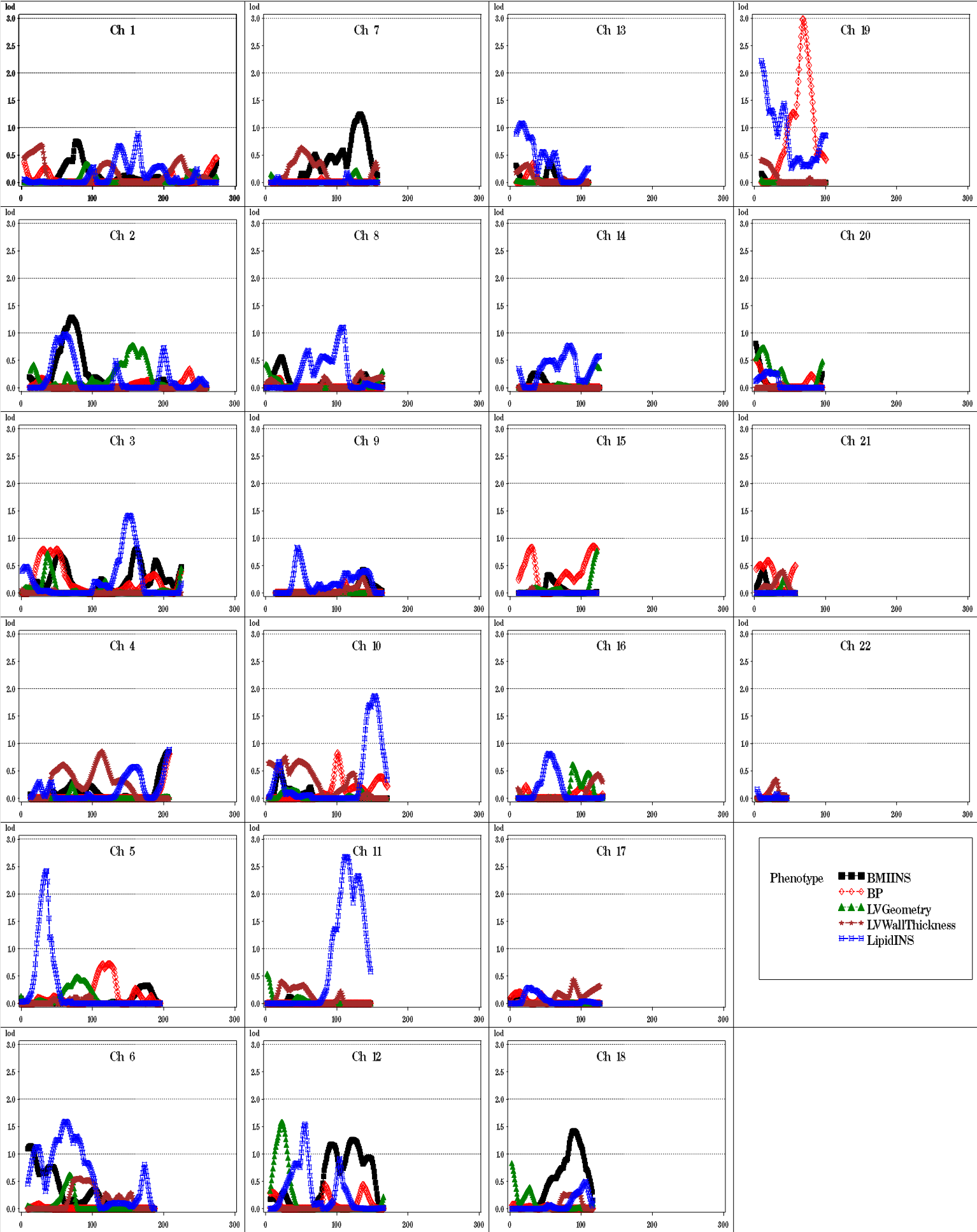

Supplement: Additional file 3 — Figure 3. Linkage analysis of factors in African Americans, all data, Varimax rotation. The graph represents the linkage analysis results. [file 1471-2350-9-103-S3.pdf]

Figure 4. Linkage Analysis of Factors, African Americans Excluding DM, Varimax Rotation

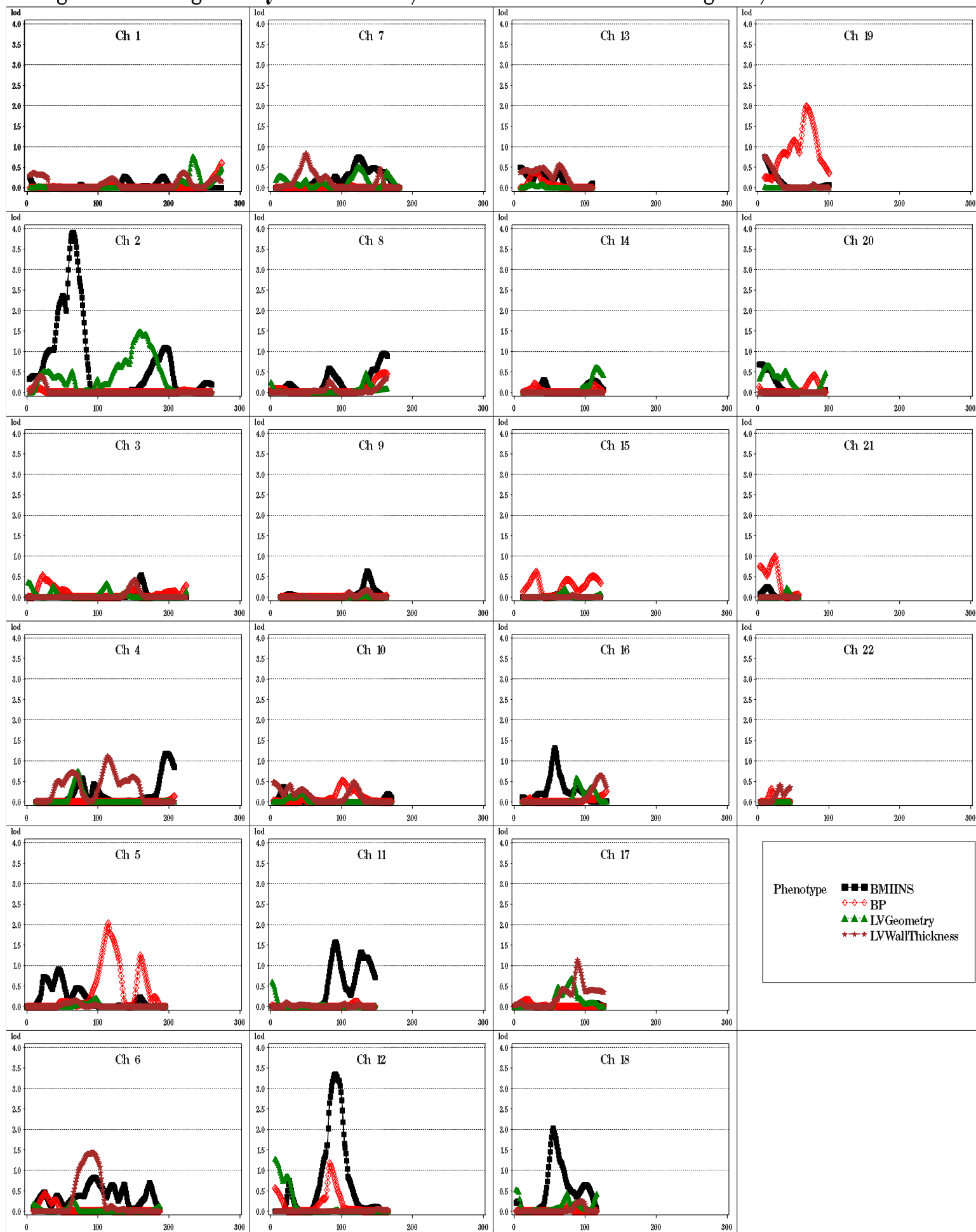

Supplement: Additional file 4 — Figure 4. Linkage analysis of factors in African Americans, excluding DM, Varimax rotation. The graph represents the linkage analysis results. [file 1471-2350-9-103-S4.pdf]

Figure 5. Linkage Analysis of Factors, Whites All Data, NO Rotation

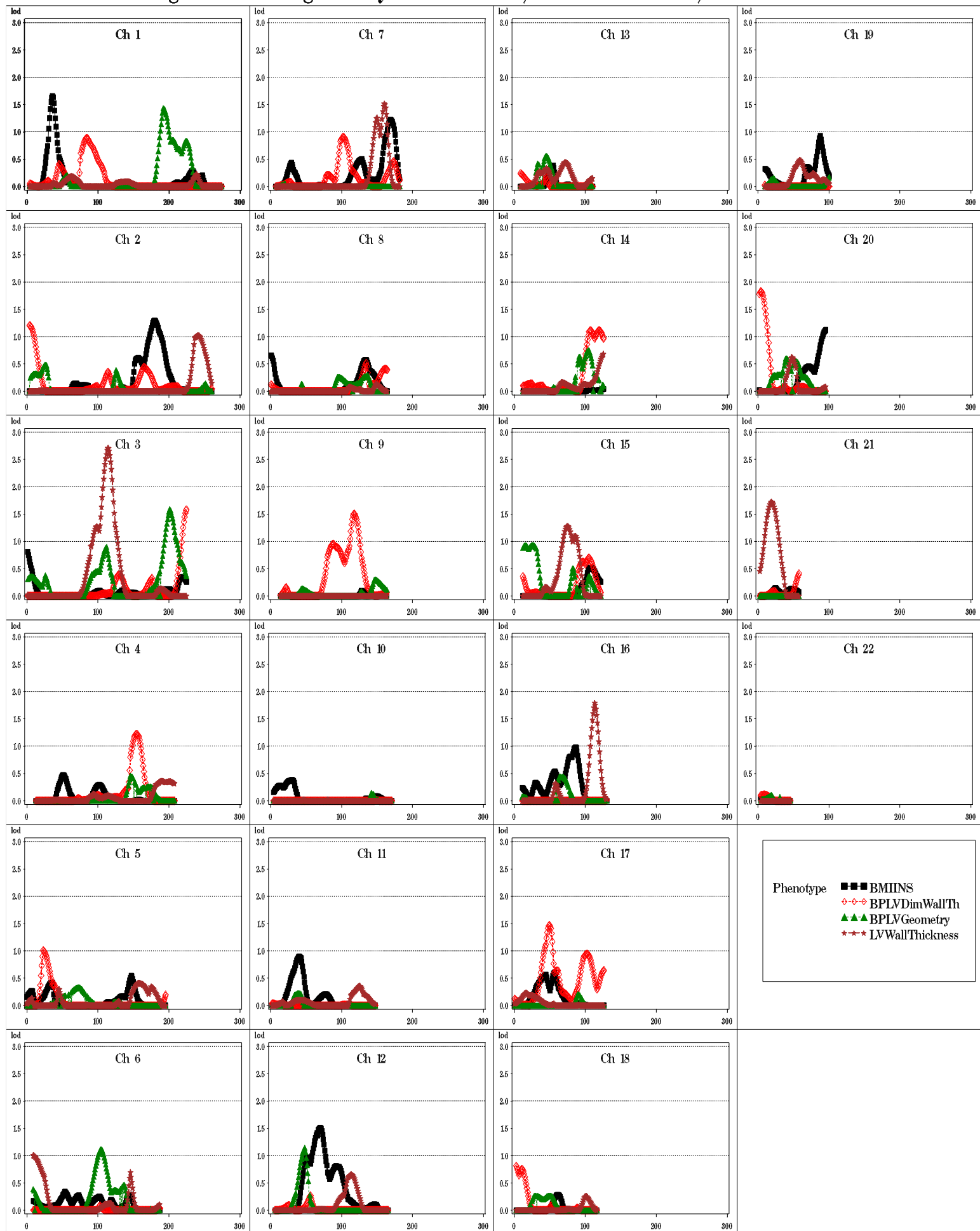

Supplement: Additional file 5 — Figure 5. Linkage analysis of factors, whites all data, no rotation. The graph represents the linkage analysis results. [file 1471-2350-9-103-S5.pdf]

# Figure 6. Linkage Analysis of Factors, Whites Excluding DM, NO Rotation

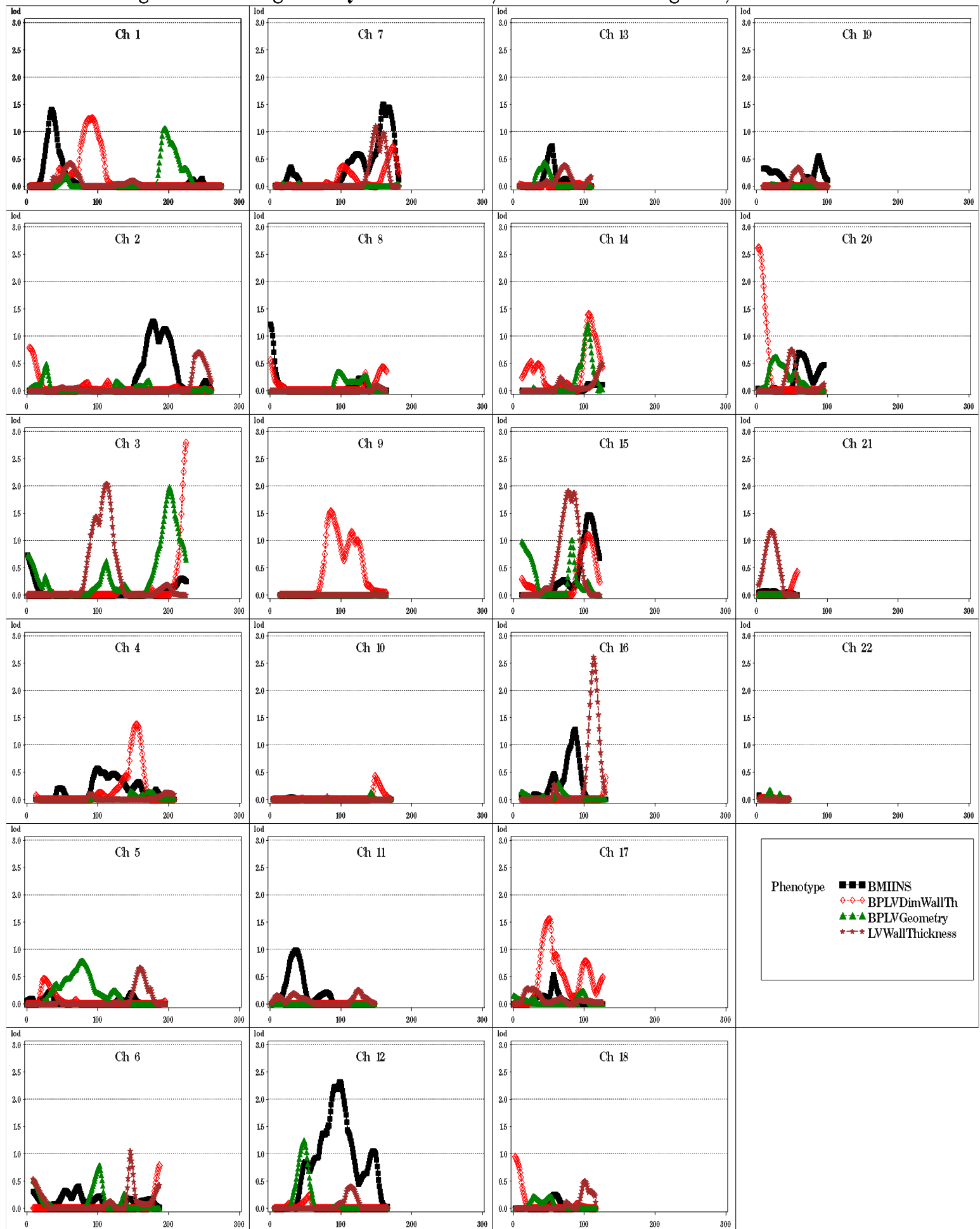

Supplement: Additional file 6 — Figure 6. Linkage analysis of factors, whites excluding DM, no rotation. The graph represents the linkage analysis results. [file 1471-2350-9-103-S6.pdf]

# Figure 7. Linkage Analysis of Factors, All Data, Varimax Rotation

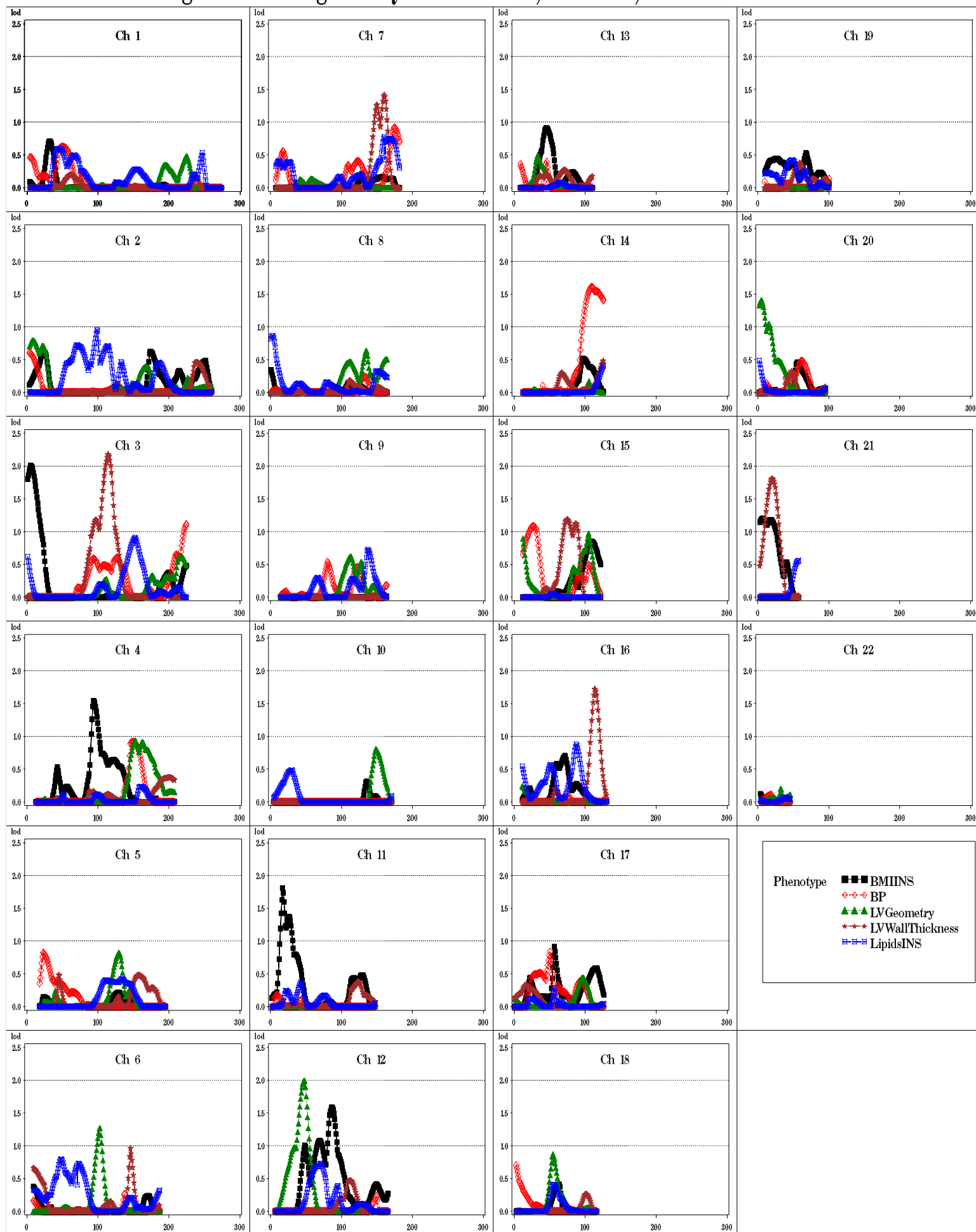

Supplement: Additional file 7 — Figure 7. Linkage analysis of factors, whites all data, Varimax rotation. The graph represents the linkage analysis results. [file 1471-2350-9-103-S7.pdf]

# Figure 8. Linkage Analysis of Factors, Whites Excluding DM, Varimax Rotation

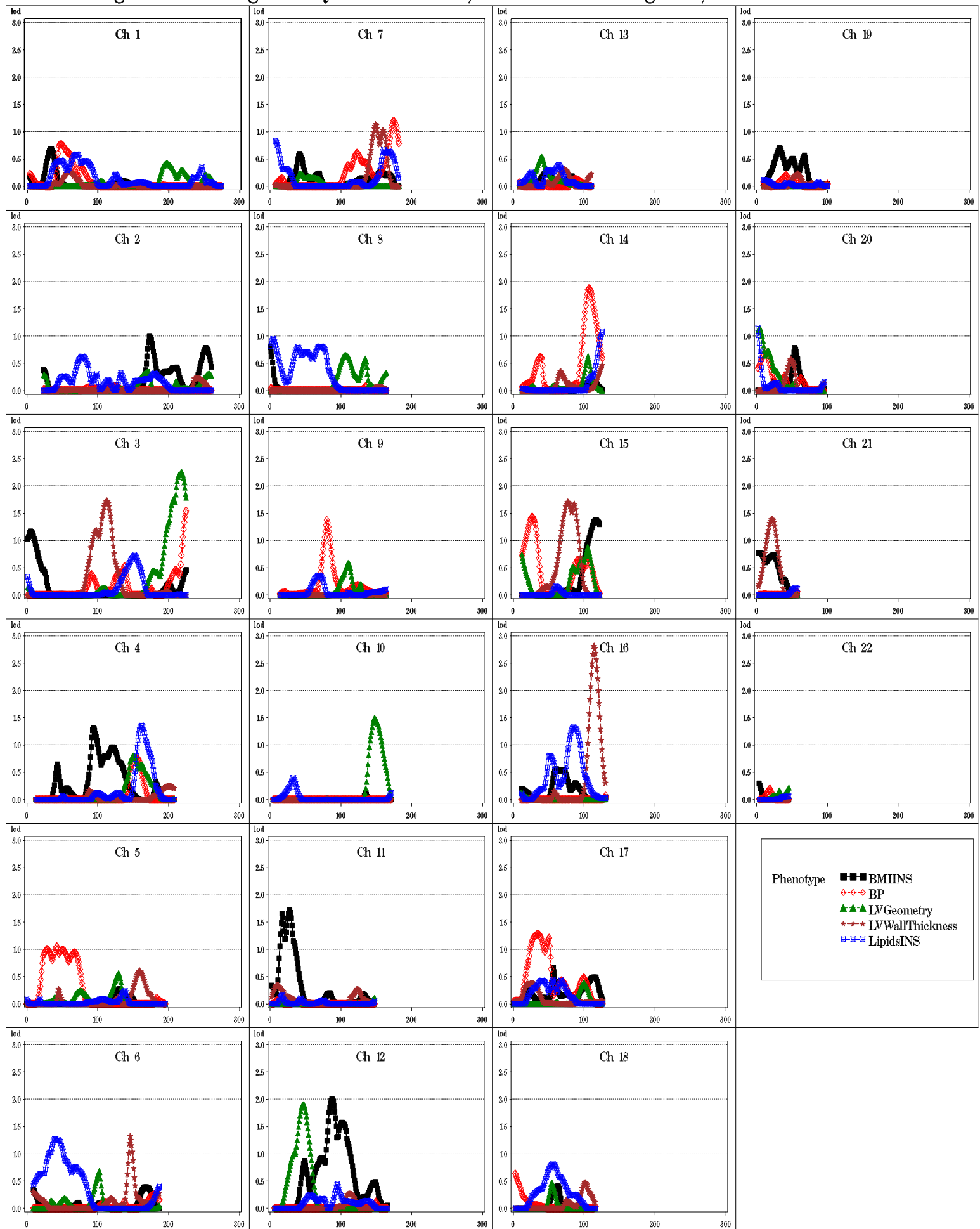

Supplement: Additional file 8 — Figure 8. Linkage analysis of factors, whites excluding DM, Varimax rotation. The graph represents the linkage analysis results. [file 1471-2350-9-103-S8.pdf]
